# Heterogeneity of cell composition and origin identified by single-cell transcriptomics in renal cysts of patients with autosomal dominant polycystic kidney disease

**DOI:** 10.7150/thno.57220

**Published:** 2021-11-01

**Authors:** Qiong Li, Yuchen Wang, Wenfeng Deng, Yanna Liu, Jian Geng, Ziyan Yan, Fei Li, Binshen Chen, Zhuolin Li, Renfei Xia, Wenli Zeng, Rumin Liu, Jian Xu, Fu Xiong, Chin-Lee Wu, Yun Miao

**Affiliations:** 1 Department of Transplantation, Nanfang Hospital, Southern Medical University, Guangzhou, China.; 2 Department of Microbiology and Infectious Disease Center, School of Basic Medical Sciences, Peking University Health Science Center, Beijing, China.; 3 Department of Pathology, Nanfang Hospital, Southern Medical University, Guangzhou, China.; 4 Department of Urology, Nanfang Hospital, Southern Medical University, Guangzhou, China.; 5 Department of Urology, Zhujiang Hospital, Southern Medical University, Guangzhou, China.; 6 Guangzhou Saliai Stem Cell Science and Technology Company Limited, Guangzhou, China.; 7 Department of Medical Genetics, School of Basic Medical Sciences, Southern Medical University, Guangzhou, China.; 8 Departments of Urology and Pathology, Massachusetts General Hospital, Harvard Medical School, Boston, U.S.A.

**Keywords:** ADPKD, Single-cell transcriptomics, Heterogeneity

## Abstract

**Rationale:** Renal cysts in patients with autosomal dominant polycystic kidney disease (ADPKD) can originate from any nephron segments, including proximal tubules (PT), the loop of Henle (LOH), distal tubules (DT), and collecting ducts (CD). Previous studies mostly used limited cell markers and failed to identify cells negative for these markers. Therefore, the cell composition and origin of ADPKD cyst are still unclear, and mechanisms of cystogenesis of different origins await further exploration.

**Methods:** We performed single-cell RNA sequencing for the normal kidney tissue and seven cysts derived from superficial or deep layers of the polycystic kidney from an ADPKD patient.

**Results:** Twelve cell types were identified and analyzed. We found that a renal cyst could be derived either from CD or both PT and LOH. Gene set variation analysis (GSVA) showed that epithelial mesenchymal transition (EMT), TNFA signaling via the NFKB pathways, and xenobiotic metabolism were significantly activated in PT-derived cyst epithelial cells while robust expression of genes involved in G2M Checkpoint, mTORC1 signaling, E2F Targets, MYC Targets V1, MYC Targets V2 were observed in CD-derived cells.

**Conclusion:** Our results revealed that a single cyst could originate from CD or both PT and LOH, suggesting heterogeneity of polycystic composition and origin. Furthermore, cyst epithelial cells with different origins have different gene set activation.

## Introduction

Autosomal dominant polycystic kidney disease (ADPKD) is mainly caused by mutations in genes polycystic kidney disease 1(PKD1) (~85%) and PKD2 (~15%) 1. It has a morbidity rate of 1/400 to 1/1000 and accounts for 5% to 10% patients with end-stage renal disease (ESRD) 2, 3.

ADPKD cysts arise from any segment of the nephron, including proximal tubules (PT), the loop of Henle (LOH), distal tubules (DT), and collecting ducts (CD) 4, 5. Tolvaptan is the only drug currently approved by FDA to treat the disease, and its target is limited to CD epithelial cells that could be one reason it does not benefit all patients 6-8. Renal cysts derived from different segments showed diverse gene expression profiles 4, 9. For example, the proinflammatory and profibrogenic factor, angiotensin, was reported to be mainly expressed in PT-derived cysts 10. Thus, the accuracy and specificity of targets are essential for drug efficacy, and identification of the origin of ADPKD cysts and molecular targets could help attenuate disease progression by developing efficient drugs.

The origin of cyst epithelial cells has not been adequately studied. It is generally believed that the origin of cells in a single renal cyst is unique 11, 12. Previous studies used limited cellular markers and failed to identify cells negative for these markers 4, 13. So, the cell composition and origin of ADPKD cyst are still unclear, and mechanisms of cystogenesis of different origins await further exploration.

Single-cell RNA sequencing (scRNAseq) is an effective way to identify the origin, composition, and differentially activated genes in various physiological and disease conditions and, to our knowledge, has not been applied to study PKD 14. This study performed scRNAseq to sequence each cell in different polycystic kidney cysts independently and steps were taken to avoid bias caused by the mixing of cells from different cysts. We investigated the cell composition in each cyst and activation of cellular signaling pathways in various cell types by scRNAseq of two normal kidney samples and seven renal cysts from an ADPKD patient. The results of our study may provide new perspectives and evidence for the hypothesis that a single cyst can originate from multiple nephron segment cell types.

## Materials and Methods

### Long-range polymerase chain reaction (LR-PCR) Sequencing

Genomic DNA was extracted from peripheral blood samples using the phenol-chloroform method. PCR of the PKD1 gene was conducted as previously reported 15, with locus-specific amplification and direct sequencing of exonic and flanking intronic regions of PKD1.

### Sample collection

The study was approved by the Nanfang Hospital Human Research and Ethical Committee (NFEC-2019-190). Seven separate cysts, greater than 2 cm in diameter each, from a freshly resected polycystic kidney, were obtained, avoiding contamination between cysts. Normal cortical and medullary parenchyma were harvested from a patient who had radical nephrectomy because of renal cancer. Single-cell suspension was obtained by trypsin and collagenase digestion.

### 10× sample processing and cDNA library preparation

The single-cell library of pretreated, qualified (cell survival rate over 80%) single-cell suspension of renal cysts was constituted according to 10× Genomics Single Cell 3′v3 Reagent Kit user guide. Beads and cells tagged with label sequence were coated in liquid drops on the microfluidic chip. Drops including single cells were collected. Cells were disrupted, permitting mRNA to bind with the cell barcode on beads to form single cell GEMs. RT reaction was done in the liquid drops, and the cDNA library was constructed after demulsification. The cell barcode was used to distinguish which cell the target sequence came from, and by the sample index on the sequence, the sample origin could be recognized. The cells and reagents were kept in one channel on the microfluidic chip and the beads in another, forming a single GEM. The reverse transcription took place independently. Subsequently, the tagged cDNA was mixed amplified, and the library was constructed. cDNA libraries of 7 cysts and 2 normal samples were successfully constructed.

### Quantification of cDNA libraries and sequencing

The molarity of each library was calculated based on its size as measured using a bioanalyzer (Agilent 2100 Bioanalyzer system) and qPCR (Quantitative Real-time PCR) amplification. For qualified libraries, samples were sequenced on Novaseq 6000 in PE150 mode with the calculated depth of ~25,000 reads per cell and 60G data measured for each library.

### scRNAseq data alignment and sample aggregating

Raw sequencing data (bel files) were converted to fastq files with Illumina bcl2fasta version 2.19.1 and aligned to the human genome reference sequence (http://cf.10xgenomics.com/supp/cell-exp/refdata-cellranger-GRCh38-1.2.0.tar.gz). The CellRanger (10×Genomics) analysis pipeline was used to generate a digital gene expression matrix from this data. The raw digital gene expression matrix (UMI counts per gene per cell) was filtered, integrated, normalized, and clustered using R package Seurat v3(https://satijalab.org/), as follows. Cells with a very small library size (< 1500 UMI counts) and a very high (> 0.3) mitochondrial genome transcript ratio were removed. Genes detected (UMI count > 0) in less than three cells were removed. As an unusually high number of genes can result from a “doublet” event, in which two different cell types are captured together with the same barcoded bead, cells with > 5500 genes were removed. The filtered gene expression matrix of all 7 samples was integrated with Seurat v3.

### Dimensionality reduction and clustering analysis

The gene expression values were subjected to library size normalization, in which raw gene counts from each cell were normalized relative to the total number of read counts of that cell. The resulting expression values were multiplied by 10,000 and log-transformed. Subsequent analyses were conducted using only the most highly variable genes in the dataset. Principal component analysis (PCA) was applied for dimensionality reduction, followed by clustering in the PCA space by a graph-based clustering approach. t-distributed stochastic neighbor embedding (t-SNE) was then used for the two-dimensional visualization of the clusters.

### Imputation of missing gene expression values

Missing gene expression values of epithelial cells were imputed using the scImpute algorithm with default parameters. Imputation was only applied to genes with dropout rates (the fraction of cells in which the corresponding gene had zero expression value) larger than 50% to avoid over-imputation.

### Differential analysis for clusters

Seurat package FindAllMarkers in Seurat v3 was used to perform differential analysis with default as function parameters. For each cluster, differentially expressed genes (DEGs) were generated relative to all other cells. We annotated each cluster from ADPKD cysts using reported cell markers (Table S1). For subsets of cells in normal samples that failed identification by a single marker, we used the Semi-supervised Category Identification and Assignment (SCINA) method 16. A gene set of epithelial subset markers specifically expressed in cyst samples (Table S1) was used. Cells were divided into a predetermined cell subset by calculating the probability. To validate the SCINA method, we also applied it to identify the cell types of the 7 kidney cyst samples and compared the results between two identification methods (cell marker vs. SCINA). The kappa coefficient reached 0.8, suggesting a good consistency between the two methods.

### Trajectory analysis

Trajectory inference Based on SNP information (TBSP) and Monocle 2 (version 2.4.0) were used for the trajectory analysis on epithelial cell populations. A set of key single nucleotide polymorphism (SNPs) was established by the TSBP method (https://github.com/phoenixding/tbsp) 17. Genes used for cell ordering were determined in an unsupervised fashion by their dispersion across cells. We selected genes with a mean expression ≥ 0.02 and an empirical dispersion parameter estimate > 1.5-fold greater than the fitted dispersion parameter estimates across the epithelial cells. Finally, trajectory construction was performed on the selected SNPs and genes with default methods and parameters.

### Calculation of ADPKD scores

The ADPKD score was used for distinguishing phenotypically modulated ADPKD epithelial cells from normal ones. The score was calculated using the highly expressed genes in ADPKD by the AddModuleScore function of Seurat v3. AddModuleScore calculated the average expression levels of each program (cluster) on the single-cell level, subtracted by the aggregated expression of control feature sets. All analyzed features were binned based on averaged expression, and the control features were randomly selected from each bin. We also downloaded the RNA-seq gene expression data of ADPKD from GEO (Gene Expression Omnibus, https://www.ncbi.nlm.nih.gov/geo/) with accession numbers GSE35831 and GSE7869. Highly expressed genes in ADPKD were calculated. The ADPKD score calculation process mentioned above was repeated.

### Gene functional annotation

Gene ontology, gene-set enrichment analysis (GSVA), and Kyoto Encyclopedia of Genes and Genomes (KEGG) pathway analysis from differentially expressed genes (DEGs) were performed using GSVA and clusterProfiler, which supports statistical analysis and visualization of functional profiles for genes and gene clusters.

### Immunohistochemistry (IHC) and immunofluorescence (IHF)

Collected tissues were fixed in 4% paraformaldehyde for dehydration, then embedded in paraffin and sliced into sections of 3 μm thickness. IHC and IF were performed according to the manufacturer's instructions. The sections were incubated with rabbit or mouse primary antibody against CD10 (GTX18040, Genetex), AQP2 (GTX31904, Genetex), LRP2 (HPA005980, ATLAS Antibodies), SLC12A1 (GTX47166, Genetex), CDH16 (ab270263, Abcam), KRT17 (ab233912, Abcam), VIM (ab92547, Abcam), NNMT (ab119758, Abcam), or pRPS6 (ab80158, Abcam) at 4 ℃ overnight. Then secondary antibody (ORIGENE) was added and incubated at room temperature for 1 h. Next, diaminobenzidine was used to reveal the color of antibody staining. Finally, the slides were mounted and observed under an optical microscope and examined histopathologically at appropriate magnification. The sections and antibodies for IHF were consistent with those used in IHC. After dewaxing, the sections were repaired in EDTA buffer (pH 8.0) at 100 ℃ and then stained with the rabbit or mouse primary antibody at 37 ℃ for 1 h. Next, secondary antibodies of corresponding species were added and incubated at 25 ℃ for 0.5 h. Finally, the sections were observed by fluorescence microscopy.

## Results

### Identification of the prime cell types in ADPKD renal cysts via scRNAseq

**Figure 1:**
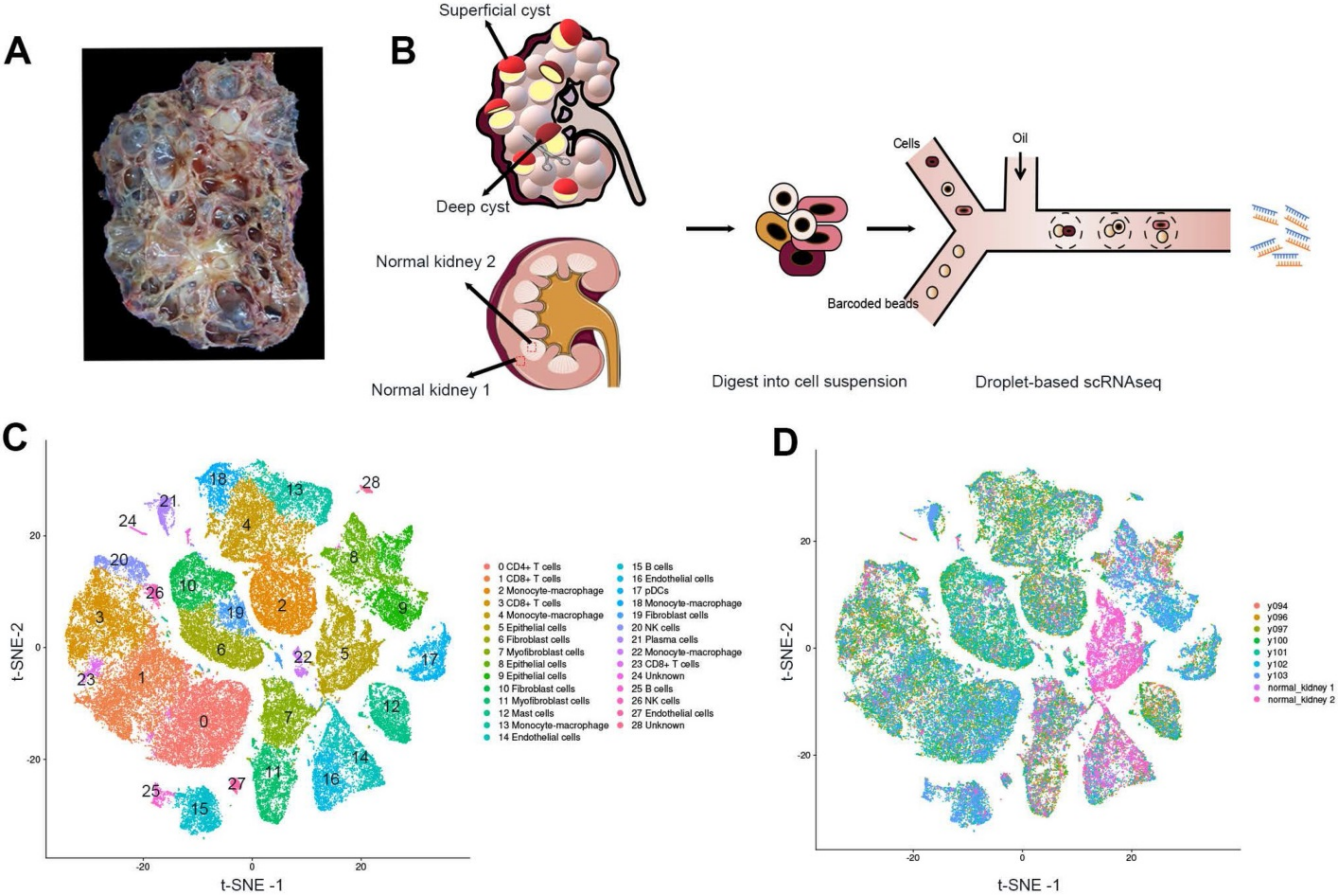
** Identification of the prime cell types in ADPKD renal cysts via scRNAseq. (A)** Gross picture of the nephrectomy specimen from ADPKD patients. **(B)** Sketch illustration of scRNAseq: the top of 5 superficial cysts and 2 deep cysts, and 2 samples from renal cortex and medulla were finely separated with surgical scissors without adhesion of surrounding tissues. Digestive enzymes were added to prepare single-cell suspension, preparing for the droplet technology-based scRNAseq. **(C)** Data of 9 samples were analyzed through the Seurat pipeline, and the distinct clusters were displayed in t-SNE projection with specific marker genes for each cluster. The precise number of each cluster is listed in Supplementary Information. **(D)** Data of 9 samples were analyzed through the Seurat pipeline, and cells are colored based on different samples. Quantitation of distinct cell clusters in each cyst sample is provided in Supplementary Information. Autosomal dominant polycystic kidney disease, ADPKD; single-cell RNA sequencing, scRNAseq; t-distributed stochastic neighbor embedding, t-SNE.

The cystic renal samples were from a 53-year-old female patient with ADPKD who suffered a repeated hemorrhage in the kidney and received nephrectomy with the preoperative estimated glomerular filtration rate (eGFR) of 8.06 mL/(min·1.73m^2^)(Figure 1A; Figure S1). LR-PCR sequencing of her peripheral blood lymphocyte DNA uncovered a frameshift mutation c.10420delC (p.Q3474Sfs*53) of the PKD1 gene, a novel mutation not yet reported in ADPKD before. The mutation resulted in an early termination at codon 53 of the polycystin-1 gene. We performed scRNAseq for seven cysts, five of which were obtained from the superficial layer (Y94, Y96, Y97, Y100 and Y101) while the other two were acquired from the deep layer (Y102 and Y103) (Figure 1B, Figure S2). Normal cortical and medullary parenchyma samples were harvested from a renal cancer patient undergoing radical nephrectomy.

Single-cell transcriptomic sequencing data of the seven cysts are available at NCBI Sequence Read Archive (SRA) with the BioProject accession number of PRJNA679848.

All the cells were divided into 12 distinct categories through unsupervised clustering analysis and specific cell markers (Figure 1C-D). Differentially expressed genes in each cluster are listed in Table S2. Sufficient epithelial cells, lymphocytes, fibroblasts, and mononuclear macrophages are obtained in the renal cysts and normal samples 18-20 (Table S3). In particular, we found that the majority of cells in 2 normal tissues were epithelial cells, and immune cells were mostly defined in 7 cystic tissues.

### Identification of the renal tubules and collecting ducts

**Figure 2:**
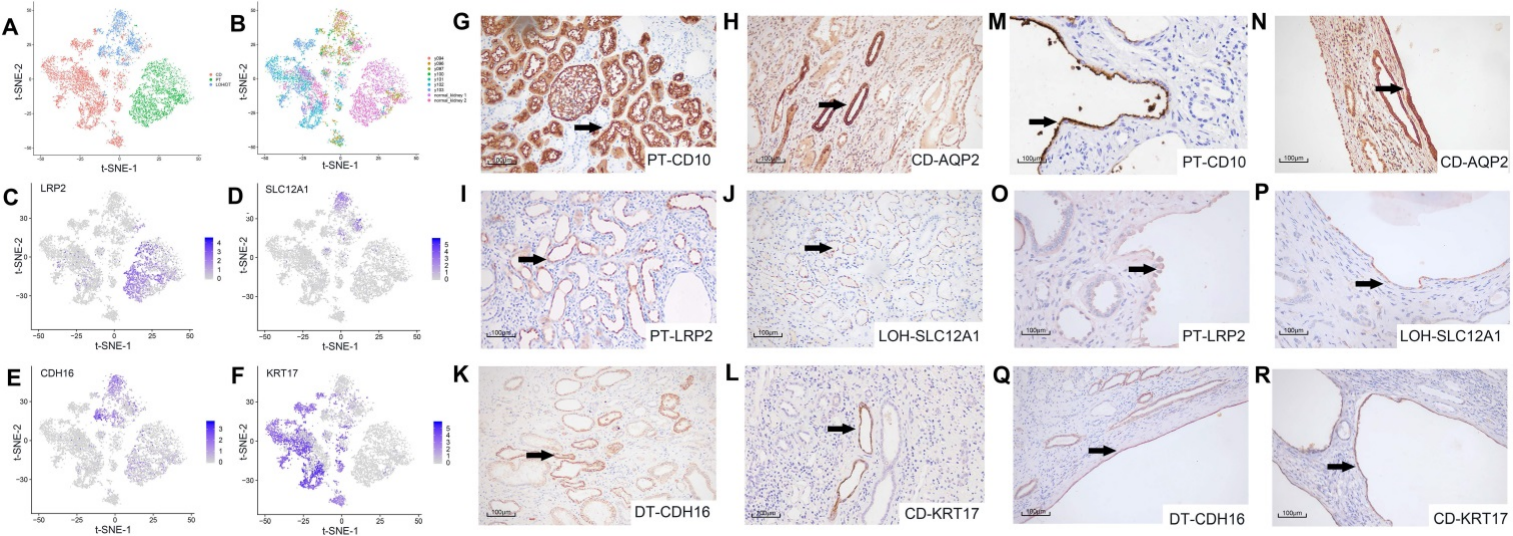
** Secondary clustering analysis of tubular cells determined 3 main clusters. (A)** Utilizing Seurat analysis, tubular cells in all samples were sorted into 3 clusters via t-SNE projection; PT-marker (+) cells are highlighted in green, LOH/DT-marker (+) in blue, and CD-marker (+) in red. **(B)** Combined t-SNE plot of defined tubular cells showing the sample source of each cluster marked by different colors. **(C-F)** Feature plots of characteristic markers for the main cell types: LRP2 (+) for PT, SLC12A1 (+) for LOH, CDH16 (+) for DT, and KRT17 (+) for CD. Expression levels are illustrated as a gradient of purple. **(G-H)** Distribution of CD10 and AQP2, classical markers of tubular cells, in normal kidney tissues. CD10 was present in PT while AQP2 in CD. **(I-L)** Distribution of LRP2, SLC12A1, CDH16, and KRT17 in normal kidney tissues. LRP2 was mainly expressed in PT and SLC12A1 was LOH shown by the black arrow. CDH16 was expressed in DT and KRT17 in CD. **(M-R)** Distribution of CD10, AQP2, LRP2, SLC12A1, CDH16 and KRT17 in polycystic kidney tissues. As the black arrow shows, CD10 and LRP2 were mainly expressed in PT and SLC12A1 in LOH. CDH16 was expressed in DT. AQP2 and KRT17 were distributed in CD. T-distributed stochastic neighbor embedding, t-SNE; proximal tubules, PT; loop of Henle, LOH; distal tubules, DT; collecting ducts, CD.

We studied the cell origin of cyst epithelial cells by conducting differential gene expression analysis and established genetic markers (LRP2, SLC12A1, CDH16, and KRT17) 21, 22 of renal tubules and CD (Figure 2A-B) in the epithelial cell populations, respectively. Epithelial cells were divided into three main clusters of PT, LOH/DT, and CD. Canonical biomarkers LRP2, SLC12A1, CDH16, and KRT17 were applied to define PT, LOH/DT, and CD (Figure 2C-F). Since cells expressing the LOH marker were also CDH16(+), LOH/DT cells were considered a combined group.

We validated the clustering outcomes by using specific antibodies for different nephron segments, including LRP2, SLC12A1, CDH16, KRT17 and two classical markers CD10 and AQP2(the marker of PT and CD, respectively) for IHC staining of cyst epithelial cells. Normal kidney tissue sections were used as a positive control. The staining results demonstrated the expression specificity of LRP2, SLC12A1, CDH16, and KRT17 in different tubular segments (Figure 2G-L). And we also identified that the cyst epithelial cells originated from PT, LOH, DT, or CD, respectively (Figure 2M-R). Similar results were obtained by IHF staining (Figure S3).

### Heterogeneity of epithelial cell composition and cell origin

We analyzed a list of previously reported genes highly expressed in ADPKD (referred to as ADPKD gene set) at the single-cell level to ascertain the gene-expression signatures of cyst epithelial cells 23.

**Figure 3:**
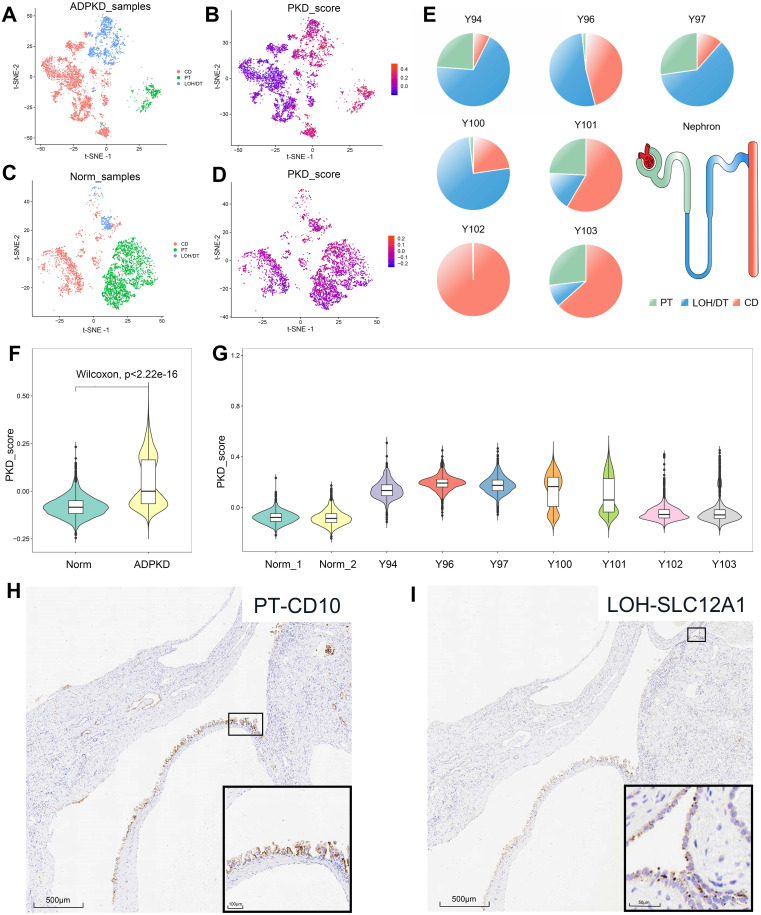
** Distinguishing the normal and cystic epithelial cells and immunohistochemical verification of multiple cellular origins in individual cyst. (A)** t-SNE plots showing the origin of epithelial cells from ADPKD tissues. Cell clusters expressing PT, LOH/DT, and CD markers were labeled as green, blue and red respectively. **(B)** t-SNE plots showing ADPKD gene set scores of epithelial cells from ADPKD tissues. **(C)** t-SNE plots showing origin of epithelial cells from normal kidney tissues. Cell clusters expressing PT, LOH/DT, and CD markers were labeled as green, blue, and red respectively.** (D)** t-SNE plots showing ADPKD gene set scores of epithelial cells from normal kidney tissues.** (E)** Origins of epithelial cells with high ADPKD gene set scores in each sample. Cell clusters expressing PT marker, LOH/DT marker, and CD marker were highlighted in green, blue and red respectively. **(F)** Violin plots showing distributions of ADPKD gene set score in normal and ADPKD tissues.** (G)** Violin plots showing distributions of ADPKD gene set score in different samples. **(H-I)** Individual cyst could derive from two cell types: serial section staining showed distribution of proximal tubule marker CD10 and LOH marker SLC12A1 in the same cyst. t-distributed stochastic neighbor embedding, t-SNE; autosomal dominant polycystic kidney disease, ADPKD; proximal tubules, PT; loop of Henle, LOH; distal tubules, DT; collecting ducts, CD.

In cystic tissues from ADPKD patients, about 50% epithelial cells showed high PKD scores, which included almost all PT- and LOH/DT-derived epithelial cells and 25% CD-derived epithelial cells (Figure 3A-B, Table S4). We also tested the data on normal tissues and found that 93% of epithelial cells were considered in the normal state based on the score, which included 97% PT-derived cells, 89% LOH/DT-derived cells and 85% CD-derived cells (Figure 3C-D, Table S4). Epithelial cells derived from superficial layers showed high PKD scores (Figure 3F-G). We also calculated the ADPKD score by integrating more published RNA-Seq data (GSE35831 & GSE7869). The results likewise suggested that a single renal cyst could originate from one cell type only or include multiple cell types (Figure S4).

Figure 3E showed the origins of epithelial cells with high PKD scores in each sample. Cysts harvested from the deep layer (Y102 and Y103) may not only be CD-derived, but could originate from 2-3 cell types. Superficial cysts, on the other hand, basically included 2-3 cell sources. To validate the results, we further performed IHC staining for PT, LOH/DT, and CD markers. The staining results proved that cells of a single cyst could be originated from a single source (CD) or from diverse sources (PT and LOH) (Figure 3H-I), providing reasonable evidence of the heterogeneity of renal cyst origin.

**Figure 4:**
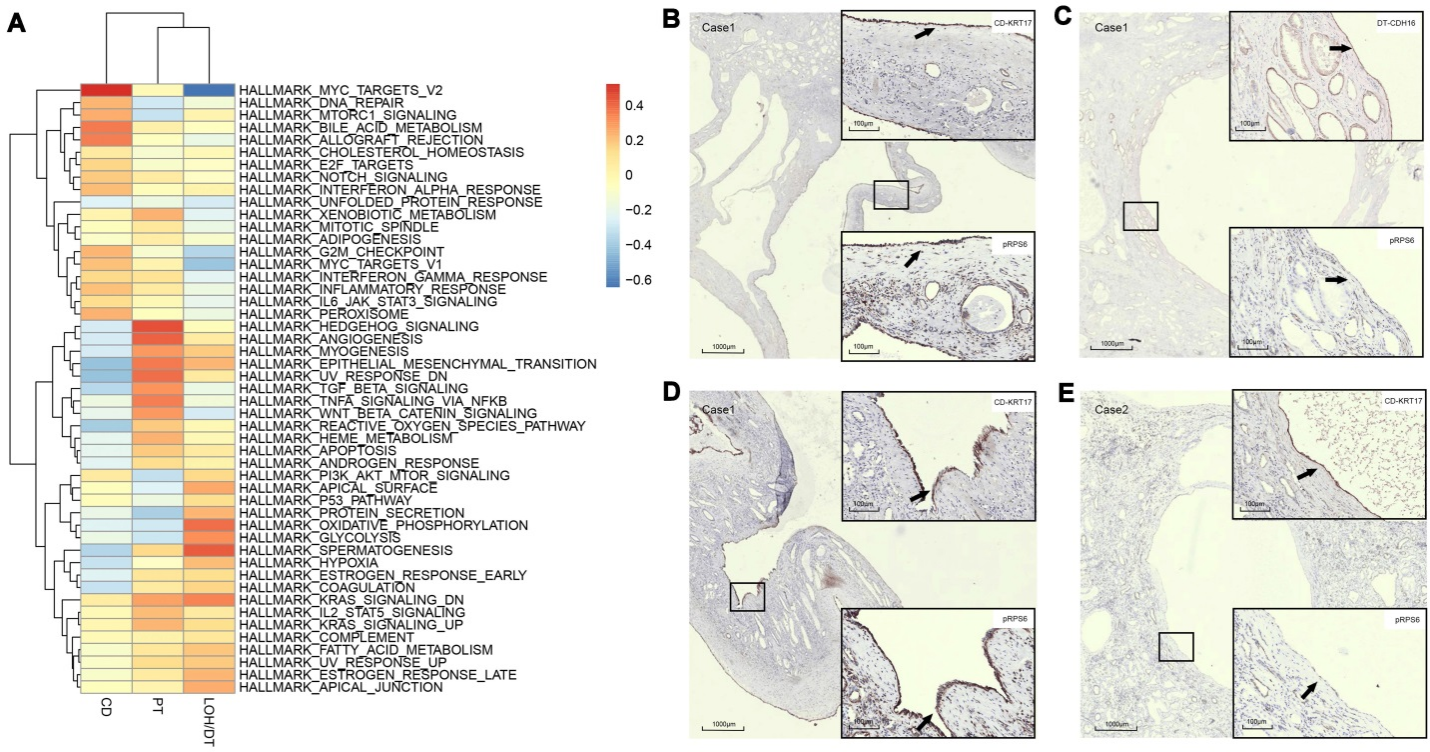
** Assessment of cyst epithelial cells diversity via GSVA. (A)** GSVA of epithelial cells segregated by cell origins. PT-derived epithelial cells demonstrated significant activation of epithelial-mesenchymal transition, TNFA signaling via the NFKB pathway, and xenobiotic metabolism pathways while robust expression of genes involved in G2M checkpoint, mTORC1 signaling, E2F Targets, MYC targets V1, MYC targets V2 and the like were observed in CD-derived cells**. (B-C)** IHC staining of CDH16, KRT17, and pRPS6 was performed in serial sections of the polycystic kidney. The results showed that the cyst from CD expressed pRPS6 protein, while the cysts from DT in the same section did not express pRPS6. **(D)** IHC staining of KRT17 and pRPS6 was performed in serial sections of the polycystic kidney. The results showed that the cyst from KRT17 (+) epithelium expressed pRPS6. **(E)** IHC staining of KRT17 and pRPS6 was performed in serial sections of the polycystic kidney. The results showed that the cyst from KRT17 (+) epithelium did not express pRPS6. Gene set variation analysis, GSVA; t-distributed stochastic neighbor embedding, t-SNE; autosomal dominant polycystic kidney disease, ADPKD; proximal tubules, PT; loop of Henle, LOH; distal tubules, DT; collecting ducts, CD; immunohistochemistry, IHC.

In order to validate the drug target, we utilized specific antibodies for mammalian phosphorylation RPS6 to perform IHC on cyst epithelial cells and found distinct expression of pRPS6 in cysts from different sources. For example, CD cysts expressed the pRPS6 protein, whereas the CDH16-positive cysts in the same section did not express pRPS6 (Figure 4B-C). The staining results also proved the difference of pRPS6 expression in CD-derived cysts (Figure 4D-E). Furthermore, the expression of EMT pathway-associated proteins of the PT-derived cysts was higher in the same sample than CD-derived cysts. As shown in Supplementary Figure S5C and S5G, PT-derived NNMT expression was higher than CD-derived NNMT. In the tissue section of another patient, this difference was observed by vimentin staining (Figure S5K).

## Discussion

Our results revealed the cellular heterogeneity in ADPKD cysts, which could be either single-origin or both PT and LOH origins. Gene set activation differed among cyst epithelial cells of different origins.

The clustering results revealed that epithelial cells and other cell types were independent of each other and that epithelial cells were divided into different subtypes because of differences in cell origin and sample source.

One of the important findings of our study was that by comparing the cell scores between the ADPKD and normal epithelial cells, we were able to hypothesized that a single cyst can either arise from CD homogeneously or from multiple cell types simultaneously. IHC staining for specific markers on serial sections of cysts (Figure 3H-I) showed a positive staining for both CD10 and SLC12A1 in a same cyst wall, suggesting PT and LOH origins, respectively. These provided a reasonable basis for heterogeneity in the composition of individual cyst.

Our findings provided new insight into ADPKD pathogenesis, indicating that there is no direct relationship between the anatomical location and cell origin of the cyst. In other words, cysts from the deep layer of the kidney do not necessarily originate from CD. The initial cysts can affect adjacent normal tubules in two aspects. First, cysts produce physical compression. For instance, a cyst with 400 μm diameter may block 32 adjacent tubules in the cortex, while a cyst with the same size occurring in the medulla can obstruct the tubule fluid from about 168,000 upstream tubules by extruding the CD 24. And second, the initial cyst may also alter the microenvironment by increasing the expression of phospho-STAT3, phospho-CREB, phospho-AKT, phospho-ERK1/2, and LCN2, thereby indirectly affecting the proliferation and apoptosis signaling in normal tubules 25, 26.

**Figure 5:**
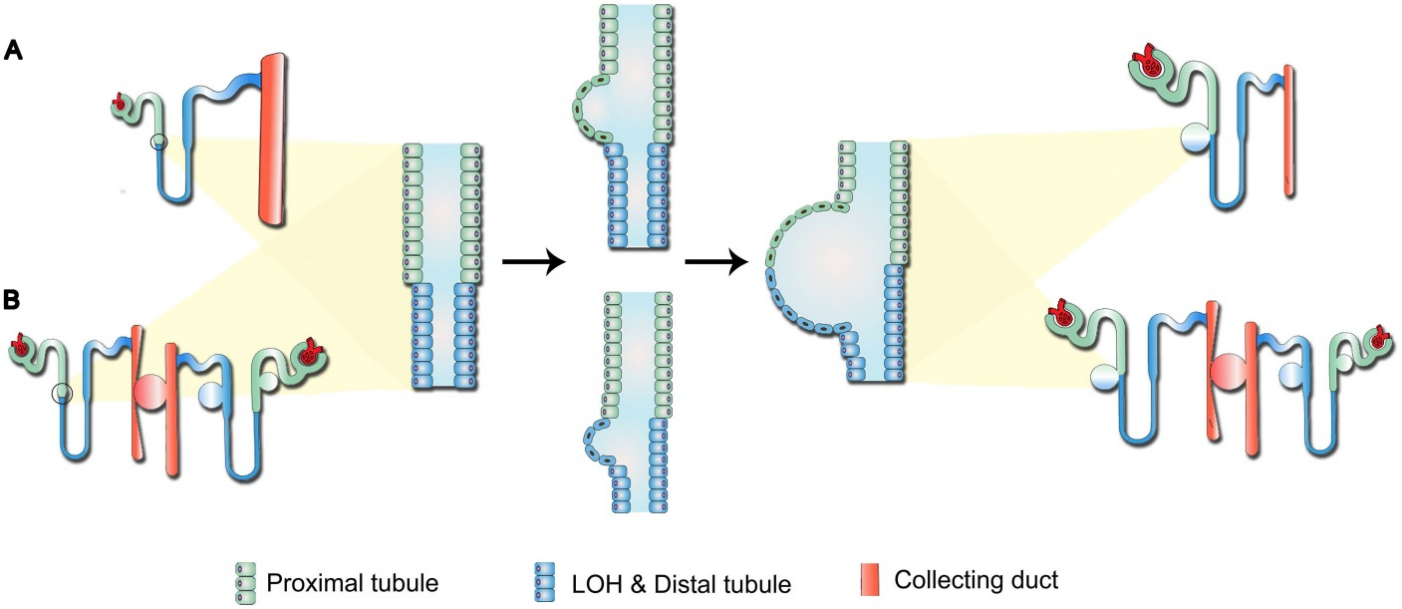
** Potential mechanism of cystogenesis. (A)** Direct formation by the synchronous or successive expansion of consecutive segments of tubules.** (B)** Indirect formation through compression of initial cysts on surrounding normal nephrons, leading to retention of tubular fluid. Over time, the growing initial cysts could completely obstruct the upstream nephrons and yield a renal cyst characterized by the composition of several types of tubular cells.

Previous studies have indicated that the continuous pressure exerted by the cyst on surrounding tissues triggers the “snowball effect”, namely locally aberrant ADPKD-related signaling could increase the possibility of new cysts formation, resulting in accelerated disease progression in the end-stage ADPKD 25. Therefore, for the cysts composed of both PT and LOH epithelial cells reported in our study (Figure 3D-E), we proposed two potential models for their development (Figure 5): first, direct formation by the synchronous or successive expansion of consecutive segments of tubules; and second, indirect formation through compression of the initial cysts on surrounding normal nephrons, leading to tubular fluid retention. Over time, the growing initial cysts could completely obstruct the upstream nephrons and yield a renal cyst characterized by the composition of several types of tubular cells.

Trajectory analysis of the epithelial cells indicated that the epithelial cells were in different states (Figure S6). We also found that the activation of dysregulated pathways commonly detected in ADPKD, such as EMT, TGF-β signal transduction, G2M, C-MYC, and mTOR signaling pathways differed among PT-, DT-, and CD-derived epithelial cells (Figure 4A). Additionally, the expression of pRPS6, which could be used to detect the activity of the mTORC1 pathway, was different in the cysts from different sources. For example, Figures 4G-H reveal that CD cysts expressed the pRPS6 protein, whereas CDH16-positive cysts in the same section did not express pRPS6 (Figure 4B-C). The staining results also provided evidence for the difference in pRPS6 expression even in CD-derived cysts (Figure 4D-E).

It has been reported that the mTOR inhibitor, rapamycin, failed to exert positive effects in impeding disease progression in ADPKD patients 27, 28. We speculated that the heterogeneity of cell origin could be the reason for the difference in drug response. Therefore, the cystogenesis mechanism in different segments should be further explored. For cysts with various cell types, combination therapy with different targets is needed to block activation of the corresponding pathways and progression of the disease.

In the present study, most PT-derived epithelial cells showed activation of the ADPKD gene set, including EMT, TNFA signaling via the NFKB pathway, and xenobiotic metabolism signaling pathways. Previous studies have demonstrated that increased activity of the EMT pathway in tubular epithelial cells is closely related to the deposition of extracellular matrix in end-stage renal fibrosis 29, 30. Besides, activation of the TNF-α pathway can lead to interstitial inflammation of the ADPKD kidney and accelerate cystogenesis. Additionally, abnormal energy metabolism, like the expedited glycolysis in PT, is related to aberrant proliferation and apoptosis in cyst epithelial cells 31. Our sequencing results also highlighted the critical role of PT in secreting cytokines like TGFB1 gene and CCL2 gene. TGF-β1, involved in the EMT process, has been shown to be significantly up-regulated in cyst epithelial cells and results in excessive extracellular matrix deposition 32, 33. Up-regulation of CCL2 in PKD1 knockout mice can promote macrophages proliferation and cysts growth by proliferation-dependent or non-dependent mechanisms 34. IHC results also showed that in some patients the expression of EMT protein was higher in PT-derived cystic epithelial cells than in CD-derived cells. Overall, while previous studies have generally suggested that the CD plays a decisive role in disease progression, our study found that activation of gene sets associated with end-stage disease was more pronounced in PT.

Although we had a sufficient number of cells to allow us to analyze the differences among cells of different origins (Table S5), based on the current analysis strategy, we were unable to distinguish the independent effects of different types of mechanisms (e.g. ischemia, surrounding fibrosis) on cells. Studies of creER-mediated models of conditional inactivation of PKD1 gene will help to further elucidate the cyst formation process during disease and are expected to reveal whether drug targets are cell-source specific.

In summary, our study is the first single-cell RNA sequencing study of ADPKD. We demonstrated that a single cyst can originate from multiple cell types. Besides, cyst epithelial cells with different origins have different gene set activation. These findings may offer novel therapeutic targets for ADPKD and validation at the histological level also provides a reasonable explanation for these findings. Nevertheless, further experimental validation studies are still needed.

## Supplementary Material

Supplementary figures and tables.

Supplementary figures and table S2.

## Abbreviations

ADPKD: autosomal dominant polycystic kidney disease
PKD: polycystic kidney disease
PT: proximal tubules
LOH: loop of Henle
DT: distal tubules
CD: collecting ducts
scRNAseq: single-cell RNA sequencing
GSVA: gene set variation analysis
EMT: epithelial-mesenchymal transition
ESRD: end-stage renal disease
LR-PCR: long-range polymerase chain reaction
SRA: sequence read archive
qPCR: Quantitative Real-time PCR
PCA: principal component analysis
t-SNE: t-distributed stochastic neighbor embedding
DEGs: differentially expressed genes
SCINA: semi-supervised Category Identification and Assignment
SNPs: single nucleotide polymorphism
TBSP: Trajectory inference Based on SNP information
GEO: Gene Expression Omnibus
KEGG: Kyoto Encyclopedia of Genes and Genomes
IHC: Immunohistochemistry
IHF: immunofluorescence
eGFR: estimated glomerular filtration rate
