# Supplementary material for: Heterogeneity of cell composition and origin identified by single-cell transcriptomics in renal cysts of patients with autosomal dominant polycystic kidney disease
Source: Theranostics. 2021 Nov 1;11(20):10064–73. doi: 10.7150/thno.57220 (PMC8581434; doi:10.7150/thno.57220)
Supplement: Supplementary file 1 — Supplementary figures and tables. [file thnov11p10064s1.pdf]

Supplementary Materials

Figure S1. Family pedigree.

Figure S2. Library quality control.

Figure S3. Immunofluorescence staining for tubular markers.

Figure S4. Cell scoring based on GSE35831 and GSE7869.

Figure S5. IHC staining for tubular markers and EMT-related protein.

Figure S6. Assessment of continuity and diversity of cyst epithelial cells via pseudo-temporal ordering analysis and GSVA.

Table S1. Epithelial cell marker list.

Table S2. Differential gene expression list.

Table S3. Quantitation of each cell type.

Table S4. Epithelial cell origin and composition of each sample.

Table S5. Quantitation of cells for the epithelial subtypes.

Figure S1. Family pedigree. Family pedigree proband (II-5) and his father, siblings (3 brothers and 1 sister) all had autosomal dominant polycystic kidney disease. I-1 died of uremia. II-1 died of cerebral infarction. II-4 died of cerebral aneurysm.

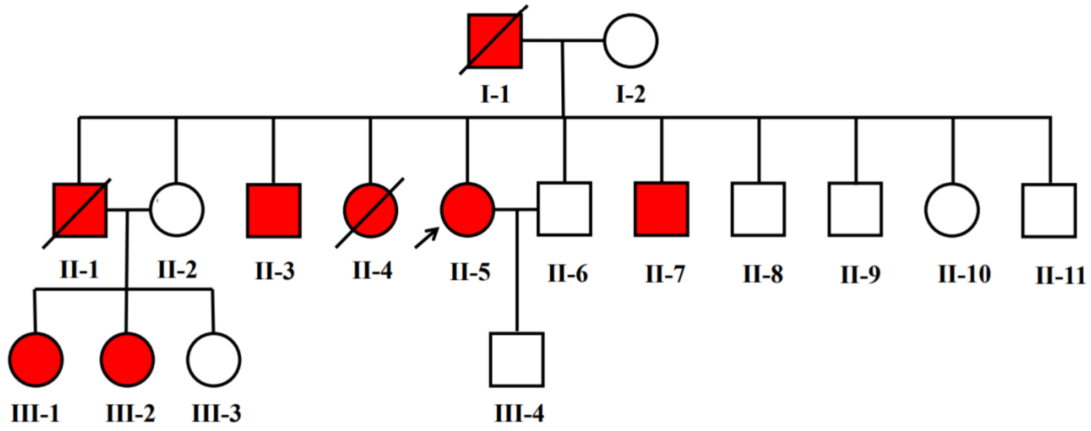

Figure S2. Library quality control. Quality control standards: appropriate size of insert segments and single peak type without spurious peak, obvious junction or primer dimer; and the concentration needs to meet the requirements of Illumina sequencing. The NGS3K assay showed that the main peak of the library was at 477-503bp (plus the splice 120bp) as shown below.

A

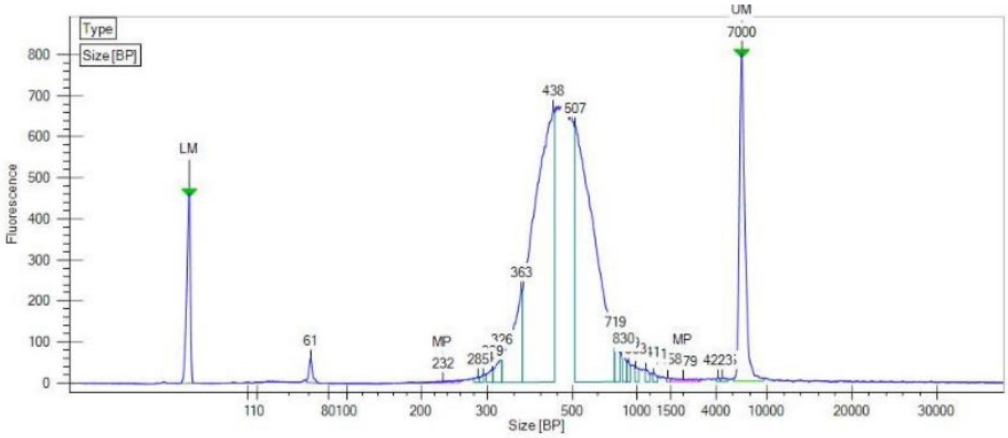

Library quality control figure _Y19SS094

B

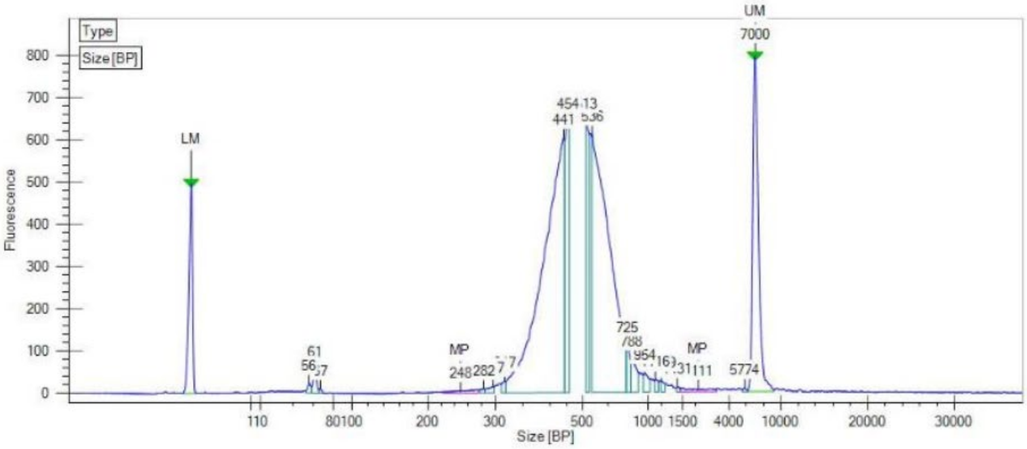

Library quality control figure _Y19SS096

C

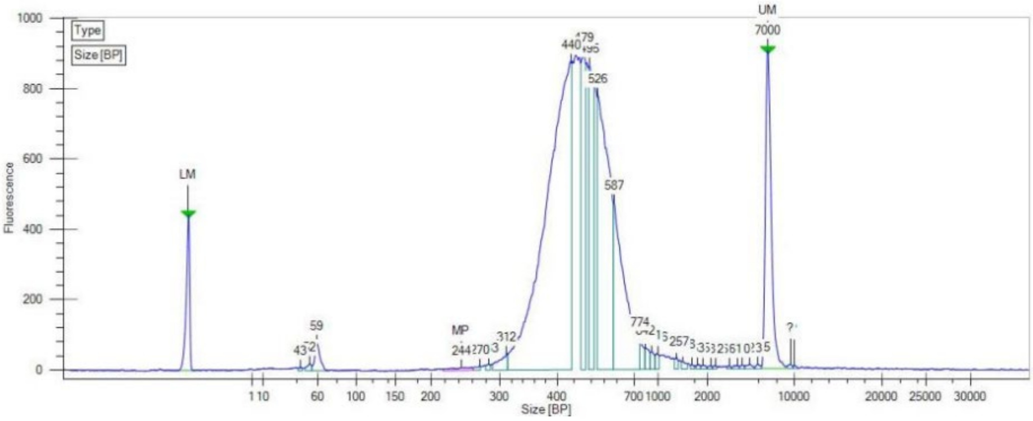

Library quality control figure _Y19SS097

D

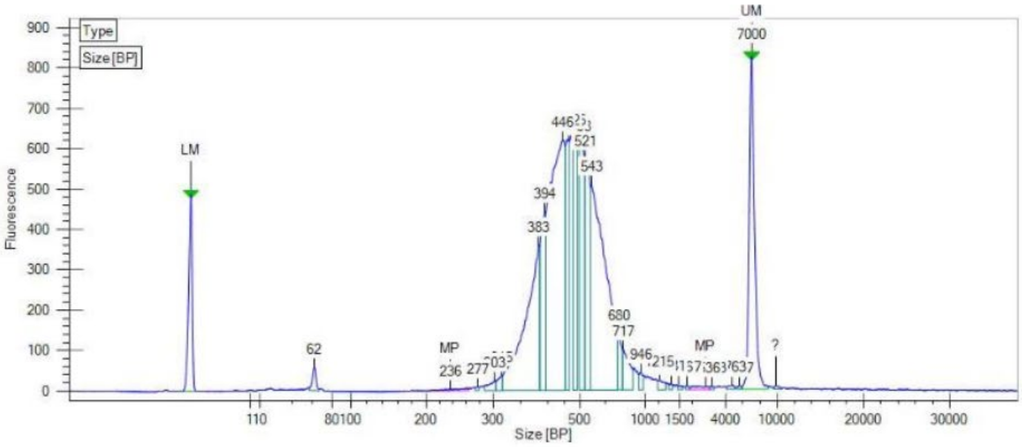

Library quality control figure _Y19SS100

E

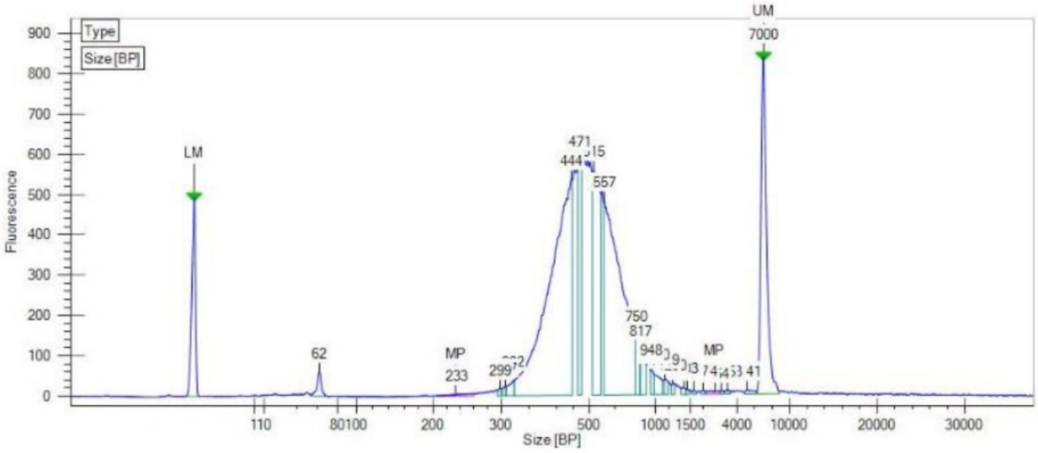

Library quality control figure _Y19SS101

F

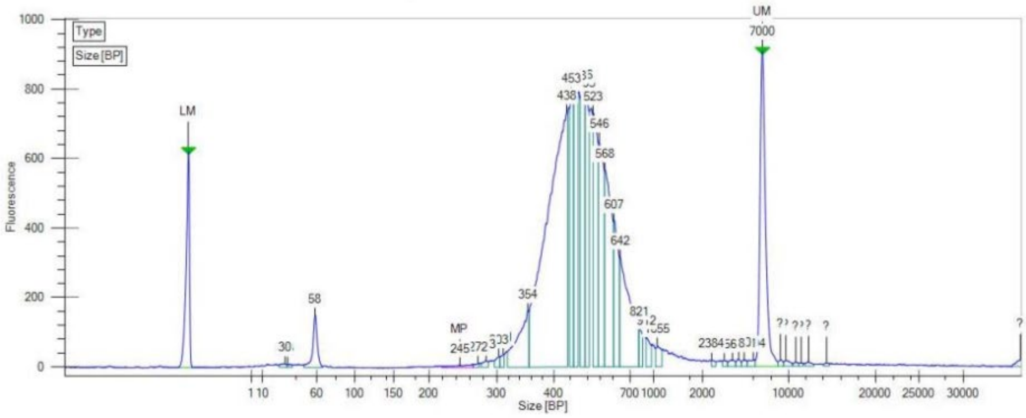

Library quality control figure _Y19SS102

G

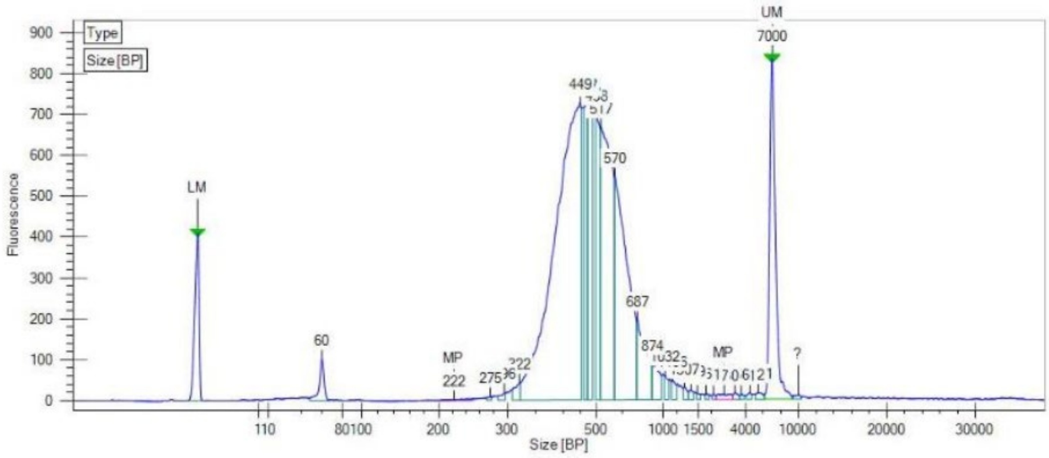

Library quality control figure _Y19SS103

H

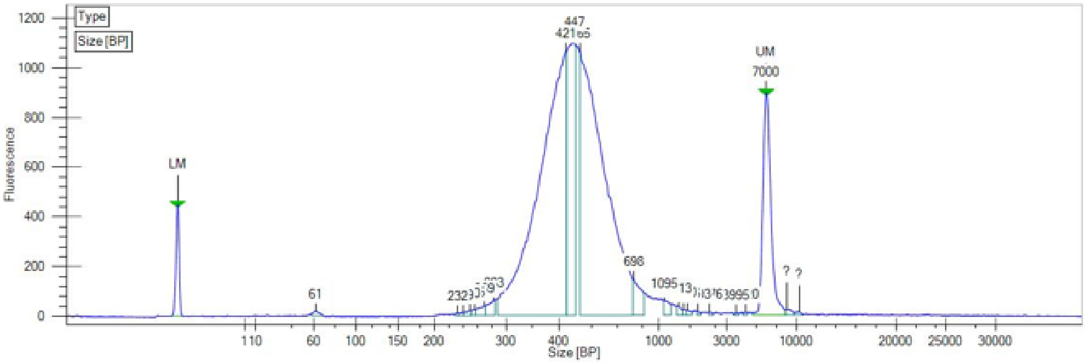

Library quality control figure-normal kidney 1

Figure S3. Immunofluorescence staining for tubular markers. (A-B) Distribution of CD10 and AQP2, classical markers of tubular cells, in normal kidney tissues. CD10 was distributed in PT while AQP2 in CD (20×). **(C-F)** Distribution of LRP2, SLC12A1, CDH16 and KRT17 in normal kidney tissues. LRP2 was mainly expressed in PT and SLC12A1 was in LOH. CDH16 gathered in DT and KRT17 in CD (20×). **(G-H)** Distribution of CD10 and AQP2, classical markers of tubular cells, in polycystic kidney tissues. CD10 was distributed in PT while AQP2 in CD (20×). **(I-L)** Distribution of LRP2, SLC12A1, CDH16 and KRT17 in polycystic kidney tissues. LRP2 was mainly expressed in PT, SLC12A1 was in LOH. CDH16 gathered in DT and KRT17 in CD (20×). PT, proximal tubules; CD, collecting ducts; LOH, loop of Henle; DT, distal tubules.

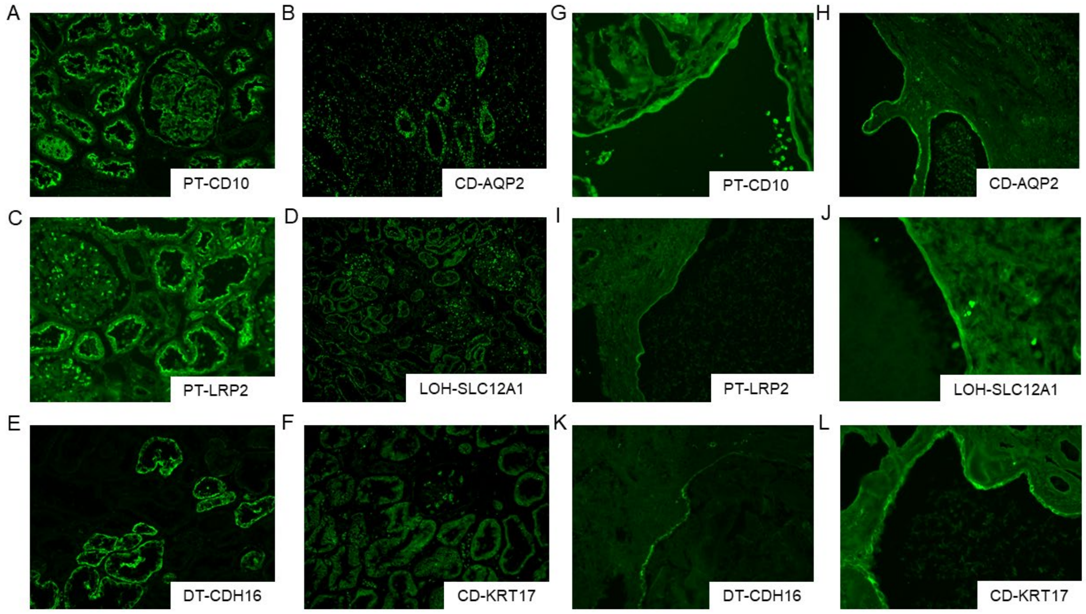

Figure S4. Cell scoring based on GSE35831 and GSE7869. (A) Combined t-SNE plot demonstrated gene scoring results in all tubular epithelial cells based on the signature genes identified from GSE35831. ADPKD gene set-high group represented diseased state while Norm group were closer to normal cells. Cell clusters expressing PT marker, LOH/DT marker, and CD marker were labeled as green, blue, and red respectively. **(B)** Combined t-SNE plot demonstrated gene scoring results in all tubular epithelial cells based on the signature genes identified from GSE7869. ADPKD gene set-high group represented diseased state while Norm group were closer to normal cells. Cell clusters expressing PT marker, LOH/DT marker, and CD marker were labeled as green, blue, and red respectively. ADPKD, autosomal dominant polycystic kidney disease; PT, proximal tubules; LOH, loop of Henle; DT, distal tubules; CD, collecting ducts.

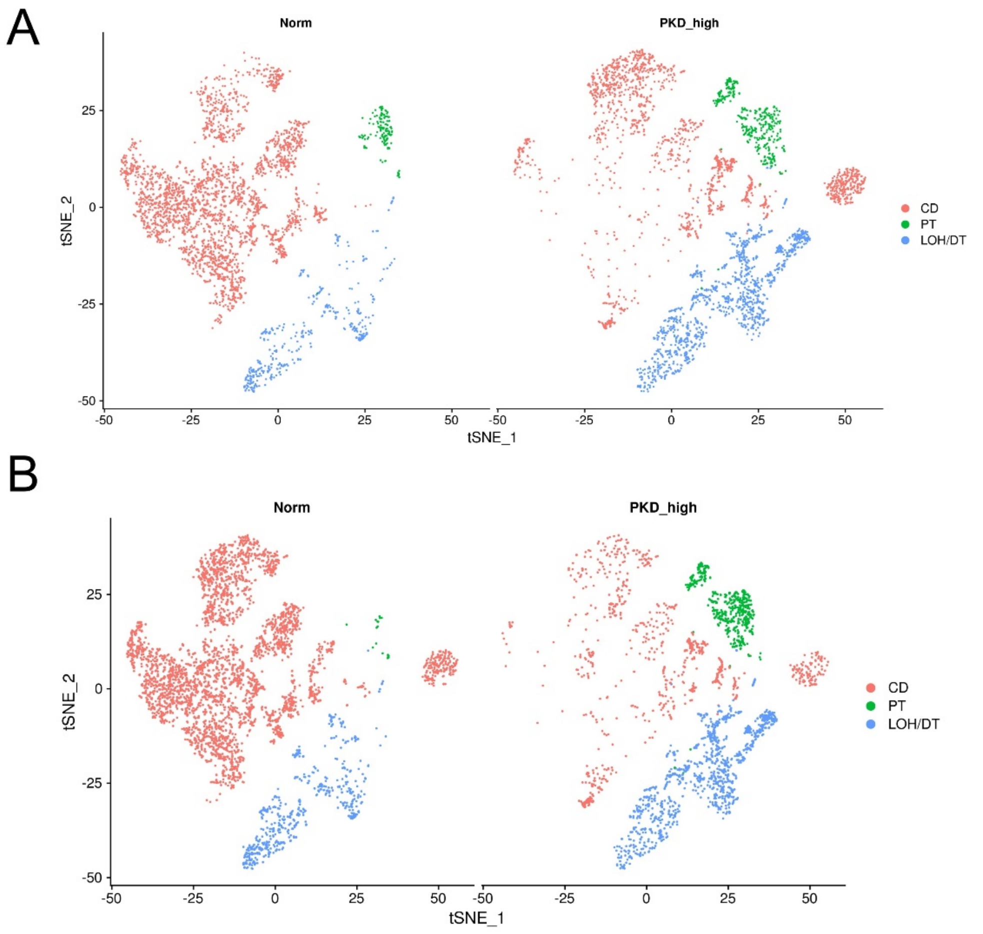

Figure S5. Immunohistochemical staining for tubular markers and EMT-related proteins. (A-D) IHC staining of LRP2, KRT17, VIM, and NMMT were performed in serial sections of tissues from polycystic kidney. **(E-H)** IHC staining of LRP2, KRT17, VIM and NMMT was performed in serial sections of tissues from polycystic kidney. **(I-M)** IHC staining of LRP2, KRT17, VIM and NMMT was performed in serial sections of tissues from polycystic kidney. Scar bar=100μm. IHC, Immunohistochemistry; PT, proximal tubules; LOH, loop of Henle; DT, distal tubules; CD, collecting ducts.

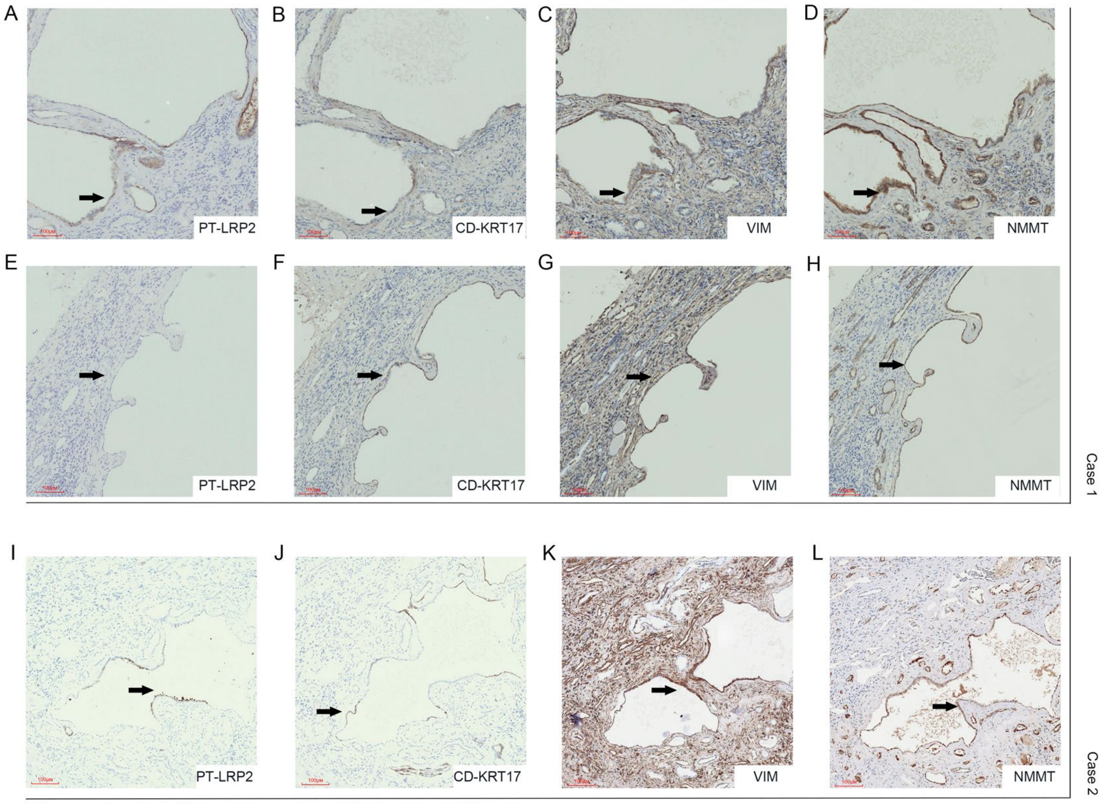

Figure S6. Assessment of continuity and diversity of cyst epithelial cells via pseudotemporal ordering analysis and gene set variation analysis (GSVA). (A) Pseudotemporal ordering was used to analyze cell trajectory and divide epithelial cells into 9 states, distinguished with different colors. **(B)** Distribution of cell clusters at different states in differentiation trajectory showed in Supplementary Figure 4A. **(C)** Distribution of epithelial cells derived from CD (red), LOH/DT (green) and PT (blue) in differentiation trajectory showed in Supplementary Figure 4A. **(D)** Assessment of the direction of differentiation trajectory based on the activation of gene sets in cells at different states. Dark blue represented the start of pseudotime. **(E)** GSVA of epithelial cells classified by cell states, in which cells at state 4, 5, 6 and 7 manifested robust expression of some of the feature ADPKD genes. GSVA, gene set variation analysis, GSVA; ADPKD, autosomal dominant polycystic kidney disease; PT, proximal tubules; LOH, loop of Henle; DT, distal tubules; CD, collecting ducts.

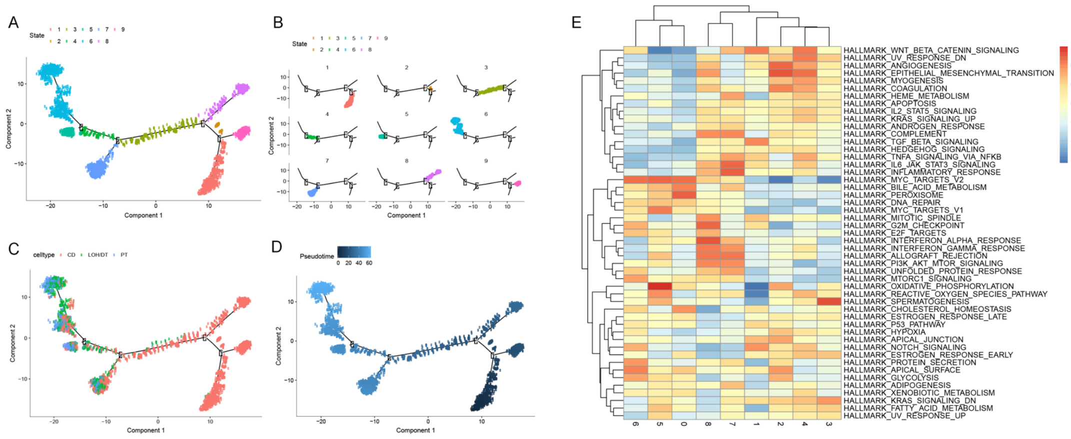

Table S1. Epithelial cell marker list

PT	LOH/DT	CD
LRP2	SLC12A1	AQP2
GPX3	TFAP2B	KRT17
GATM	UMOD	AVPR2
PDZK1	CDH16	KLF5
MME	DEFB1	EHF
		AQP3
		WNT7B
		TMEM2B
		KCNJ1
		AVPR2
		CLDN4
		ATP6V0D2

Table S3. Quantitation of each cell type

	Normal_kidney 1	Normal_kidney 2	y094	y096	y097	y100	y101	y102	y103
CD4+ T cells	39	105	732	825	846	1474	577	2001	1141
CD8+ T cells	116	309	1738	2088	1445	2381	893	1473	1049
Monocyte-macrophage	121	311	1601	3105	2080	2729	1441	1463	1179
Epithelial cells	1736	2563	774	409	793	707	501	2013	1583
Fibroblast cells	26	107	1105	1331	470	755	3230	467	285
Myofibroblast cells	119	679	1009	483	1412	428	589	356	701
Mast cells	0	4	832	425	419	397	79	202	191
Endothelial cells	438	1035	798	176	431	141	798	235	503
B cells	58	34	224	72	193	217	181	752	761
pDCs	6	1	257	177	116	294	79	550	297
NK cells	6	49	150	246	156	365	84	238	148
Plasma cells	0	0	3	39	6	380	5	45	489
Unknown	24	35	139	53	89	116	70	30	64

Table S4. Epithelial cell origin and composition of each sample

	Norm(PKD)	PKD_high
y094_CD	8	56
y094_LOH/DT	1	521
y094_PT	8	180
y096_CD	6	185
y096_LOH/DT	0	211
y096_PT	0	7
y097_CD	6	90
y097_LOH/DT	0	482
y097_PT	1	214
y100_CD	169	122
y100_LOH/DT	1	405
y100_PT	0	10
y101_CD	195	179
y101_LOH/DT	0	53
y101_PT	0	74
y102_CD	1698	314
y102_PT	0	1
y103_CD	1299	180
y103_LOH/DT	0	28
y103_PT	0	76
normal_kidney 1_CD	207	27
normal_kidney 1_LOH/DT	33	6
normal_kidney 1_PT	1425	38
normal_kidney 2_CD	949	172
normal_kidney 2_LOH/DT	210	24
normal_kidney 2_PT	1177	31

Table S5. Quantitation of cells for the epithelial subtypes

	CD	LOH/DT	PT
NORM	1355	273	2671
PKD	4507	1702	571
